# The Complete Chloroplast Genomes of Two *Lespedeza* Species: Insights into Codon Usage Bias, RNA Editing Sites, and Phylogenetic Relationships in Desmodieae (Fabaceae: Papilionoideae)

**DOI:** 10.3390/plants9010051

**Published:** 2019-12-31

**Authors:** Yamuna Somaratne, De-Long Guan, Wen-Qiang Wang, Liang Zhao, Sheng-Quan Xu

**Affiliations:** 1 College of Life Sciences, Shaanxi Normal University, Xi’an 710062, China; yamuna@snnu.edu.cn (Y.S.); guandelong@snnu.edu.cn (D.-L.G.); 2 College of Life Sciences, Yan’an University, Yan’an 716000, China; wenqiangwang@yau.edu.cn; 3 College of Life Sciences, Northwest A & F University, Yangling 712100, China; biology_zhaoliang@126.com

**Keywords:** *Lespedeza*, desmodieae, papilionoideae, fabaceae, chloroplast genome, phylogenetic relationships

## Abstract

The genus *Lespedeza* (tribe: Desmodieae) consists of about 40 species that have high medicinal and economic value. However, in this genus, using morphological characters, the species identification is quite complicated, which can be solved by the analysis of the complete chloroplast genomes. As primary organelle genomes, the complete genome sequences of chloroplasts (cp) provide unique molecular information to study the divergence of species, RNA editing, and phylogeny. Therefore, to the best of our knowledge, for the first time, we sequenced the complete cp genomes of two representative *Lespedeza* species: *Lespedeza davurica* and *Lespedeza cuneata*. The cp genomes of both the species were found to be 149,010 bp in length, exhibiting the typical angiosperm chloroplast structure containing four regions. The *Lespedeza* cp genomes showed similar conserved gene contents, order, and orientations with a total GC content of 35.0%. A total of 128 genes, including 83 protein-coding genes, 37 tRNAs, and eight rRNAs, were identified from each genome. Unique molecular features of the two *Lespedeza* cp genome sequences were obtained by performing the analysis of repeats, sequence divergence, codon usage, and predicting the RNA editing sites in addition to phylogenetic analysis with other key genera in tribe Desmodieae. Using the two datasets, the phylogenetic relationship of *Lespedeza* species among Deasmodieae was discovered, suggesting that whole cp genomes provided useful information for phylogenetic studies of these species.

## 1. Introduction

*Lespedeza* is a genus in the tribe Desmodieae, which belongs to the subfamily Papilionoideae of the family Fabaceae, and this genus comprises nearly 40 species distributed mainly in eastern Asia and eastern North America [1]. These plants grow as shrubs or trailing vines and can be either annual or perennial. Due to the structure of the plants and their inflorescences, some species like *Lespedeza bicolor*, are used as ornamental plants. The leaves of these plants can be used as forage and for soil enrichment, and its deep root system is useful to prevent soil erosion. For example, *Lespedeza cuneata* and *Lespedeza davurica* plant as a forage crop for livestock and are used to conserve soil and improve soil conditions [2,3]. Due to its high tannin content at maturity, animals are fed on *L. cuneata* at an early stage of the plant. The high adaptability to drought and good forage quality of *L. davurica* are important for its use as a fodder legume [4]. Similar to most other legumes, the deep root system of the *Lespedeza* has nitrogen-fixing nodules that harbor the symbiotic soil bacteria, making this plant a nitrogen fertilizer [5]. Moreover, several bioactive compounds that have antidiabetic activities have been extracted from some species of *Lespedeza* that were used in medicine to prevent diabetes in ancient times. Recent studies have shown that *L. davurica* exerts a protective function on β-cells in the pancreas, which can regulate blood glucose in Type 1 diabetes [6]. Due to the distinct values of each *Lespedeza* species, it is vital to differentiate them at the species level for proper utilization and conservation.

Classification of these *Lespedeza* species has been performed for many years using different methods. Ohashi [7] classified genus *Lespedeza* into two subgenera, *Lespedeza* and *Macrolespedeza*, based on the floral characters and the pollen morphology [8]. *Macrolespedeza* is distributed only in East Asia while *Lespedeza* is distributed in both eastern North America and East Asia. The identification of eastern Asian and eastern North American species based on morphological characteristics was discussed decades ago [9]. However, Ohashi and Nemoto (2014) [10] showed that these two subgenera differ only in seedling morphology. Phylogenetic analysis has been performed from the DNA sequences of the nuclear ribosomal internal transcribed spacer (ITS) and plastid regions of *Lespedeza* species [1]. Moreover, Kim et al. (2012) [11] classified *Lespedeza* species using their metabolite profiling; previous research has shown that the floral morphology of *Lespedeza* was similar to that of two genera, *Campylotropis* and *Kummerowia*, which were formerly placed in *Lespedeza* [8]. Therefore, an accurate method of plant identification is essential for *Lespedeza* species. The whole chloroplast genome contains more effective molecular markers, referred to as DNA barcodes, which are useful for identifying species accurately. Chloroplast genomic sequences provide molecular information that is a good resource for plant systematics, phylogenetic studies, and population genomics [12,13]. Therefore, in recent years, many chloroplast genomes in the family Fabaceae, have been completely sequenced. Complete chloroplast sequences of *L. maritima*, classified in subgenus *Macrolespedeza* of the genus *Lespedeza*, has been reported recently [14].

The typical angiosperm chloroplast genome is a circular, double-stranded DNA molecule with a size ranging from 120 to 160 kb [15]. It contains about 130 genes within the regions of two identical copies of inverted repeats (IRs), a large single-copy (LSC) region, and a small single-copy (SSC) region [16,17]. Chloroplast genomes are maternally inherited, and they contain highly conserved gene structures [18]. Because of its stable structures and uniparental inheritance, it has been used in comparative analysis to understand the evolution of closely related species. Over the past few years, whole chloroplast (cp) genome sequencing with next-generation sequencing technology (NGS) has become much more accurate, less expensive, less labor-intensive, and less time consuming than conventional Sanger sequencing [19,20,21]. Illumina-based NGS technology has been widely used recently, and the molecular structures of many plant cp genomes have been discovered in detail within a short period [22]. Chloroplast genomic information has been widely used for molecular marker development in different evolutionary studies [17,23]. Polymorphic simple sequence repeats (SSR) identified in the cp genomes were previously used for species identification, population genetics, and phylogenetic studies [24,25]. Codon usage bias in cp genomes has been studied as it plays an important role in gene expression [26,27]. Furthermore, identifying the RNA editing sites in the cp genome is vital to understand the correct translation process and mutations of genes [28,29]. Comparative analyses of cp genomes based on these variations are useful resources to investigate the evolutionary relationship among species.

In this study, we obtained the complete cp DNA sequences of two *Lespedeza* species in subgenus *Lespedeza*, *Lespedeza davurica* and *Lespedeza cuneata*, using an Illumina HiSeq X Ten sequencing platform. Our aims of this study were (1) to explore the molecular features of each genome compared them with the *Macrolespedeza* species, *L. maritima*, and other members of the tribe Desmodieae; (2) to observe the variable regions between species in the genus *Lespedeza*; (3) to examine the SSR distributions, codon usage patterns, and RNA editing sites among *Lespedeza* cp genomes; and (4) to determine phylogenetic position of subgenus *Lespedeza* in the tribe Desmodieae. The results obtained from this study will provide a deeper understanding and classification of this genus in tribe Desmodieae.

## 2. Results

### 2.1. Comparison of Lespedeza cp Genome Structures

**Figure 1:**
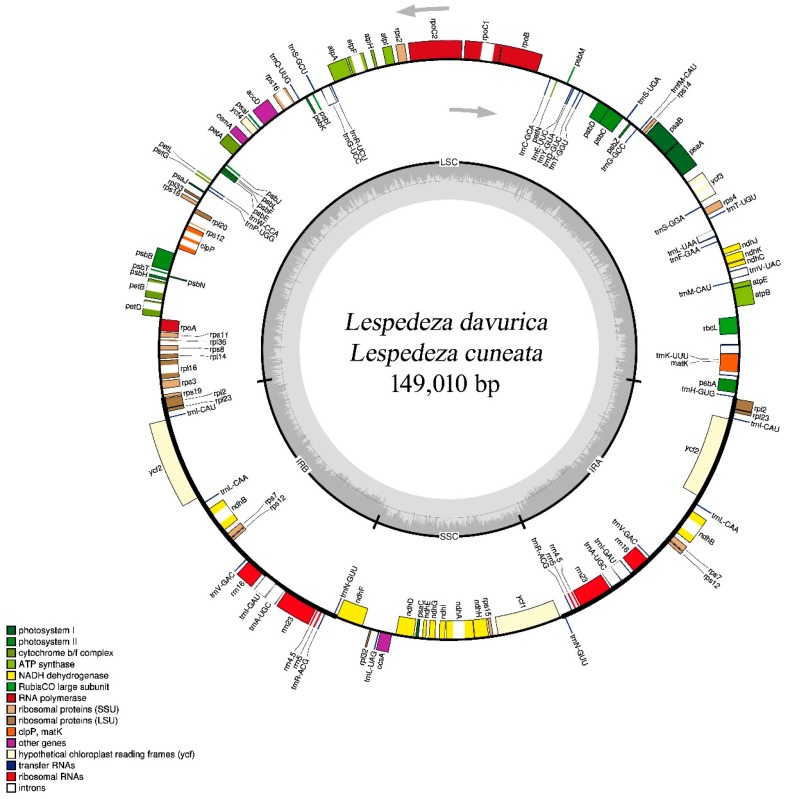
Gene map of *Lespedeza davurica* and *Lespedeza cuneata* chloroplast genomes. Genes on the inside of the map are transcribed in the clockwise direction and genes on the outside of the map are transcribed in the counterclockwise direction. The darker gray in the inner circle represents GC content whereas the light gray corresponds to AT content. Different functional groups of genes are shown in different colors. The figure was drawn with OrganelleGenomeDRAW [30].

The length of the complete genomes of both *L. davurica* (MH800328) and *L. cuneata* (MN268503) were 149,010 bp and exhibited the typical angiosperm circular chloroplast structure containing four regions: Large single-copy region (LSC; 82,373 bp, 82,395 bp), small single-copy region (SSC; 18,975 bp, 18,951 bp), and a pair of inverted repeats (IR) each (23,831 bp, 23,832 bp). The cp genomes showed similar gene contents, orders, and orientations (Figure 1). Nearly 52.2% of the complete chloroplast genomes consisted of protein-coding genes (77,868 bp), 6.1% consisted of rRNAs (9062 bp), and 1.8% consisted of tRNAs (2794 bp) in both species. In addition, similar characteristic features of the cp genomes were shared between *L. davurica* and *L. cuneata*.

The analysis revealed that the GC content of the cp genome of both the species was 35.0% and the IR regions exhibited highest GC content (42.2%) followed by the LSC regions (32.5%) and SSC regions (28.1%). The coding sequences (CDS) showed a similar total GC content of 36.0% in both species (Table 1).

A total of 128 total genes, including 83 protein-coding genes, 37 tRNAs, and eight rRNAs were identified from each genome and annotated using Dual Organellar GenoMe Annotator (DOGMA). In addition, 16 functional genes, including five protein-coding genes (*rpl2*, *rpl23*, *ycf2*, *ndhB*, and *rps7*), seven tRNA genes (*trnI-CAU*, *trnN-GUU*, *trnR-ACG*, *trnA-UGC*, *trnI-GAU*, *trnV-GAC*, and *trnL-CAA*), and four rRNA genes (*rrn16*, *rrn23*, *rrn4.5*, and *rrn5*) were duplicated in the IR regions of each chloroplast genome.

Eight protein-coding genes (*petB*, *petD*, *atpF*, *ndhB*, *ndhA*, *rpoC1*, *rpl16*, and *rps16*) and eight tRNA genes contained one intron, while three protein-coding genes (*rps12*, *ycf3*, and *clpP*) contained two introns (Supplementary Materials, Table S1). The 5′ end of the *rps12* gene was located in the LSC region and 3′ end duplicated in the IR regions in both the species. The basic features of these two chloroplast genomes were compared to six other species in the tribe Desmodieae: *Lespedeza maritima*, *Kummerowia striata*, *Campylotropis macrocarpa*, *Hylodesmum podocarpum*, *Desmodium heterocarpon*, and *Ohwia caudata* (Table S2).

### 2.2. Codon Usage Bias and Prediction of RNA Editing Sites

**Figure 2:**
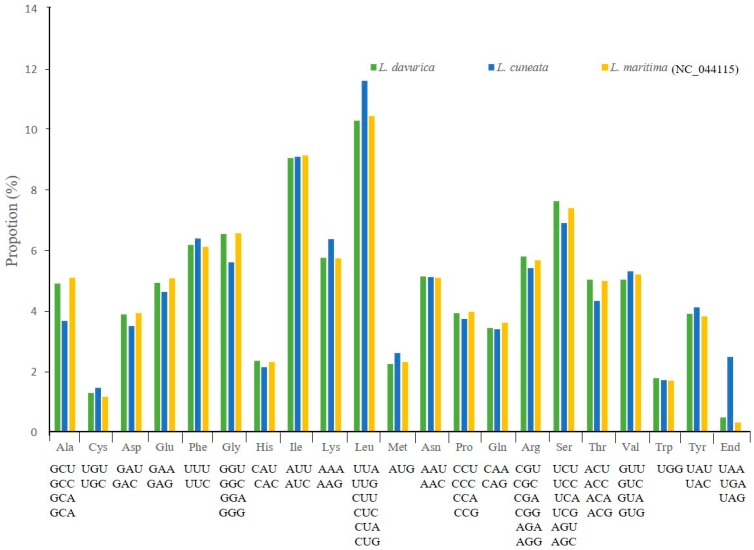
Amino acid frequencies of the chloroplast genomes of *Lespedeza* species.

We analyzed the codon distribution among all protein-coding genes in the two *Lespedeza* species and compared them with *L. maritima*. These protein-coding sequences represented a total of 25,956 codons in *L. davurica* and *L. cuneata* each and 25,958 codons in *L. maritima*. They belonged to 61 codon types, which encoded 20 amino acids. Leucine was the most abundant amino acid, whereas cysteine showed the least abundance in three species (Figure 2). The most often used synonymous codon was AAA, encoding lysine, and the least used was CGG, encoding arginine (Table S3). Relative synonymous codon usage (RSCU) is a good indicator to measure codon usage bias in coding sequences. This value is the ratio between the observed frequency and expected frequency of a codon. When the use of synonymous codons is less frequent than expected, the RSCU value is less than 1. However, when this value is more than 1, the expected frequency is less. Methionine and tryptophan showed RSCU = 1, indicating that codons AUG and UGG had no bias and preferences. The highest RSCU value was for UUA (~2.06) in leucine, and the lowest was GGC (0.3) in glycine. Moreover, leucine showed A or T (U) bias in all synonymous codons: UUA, UUG, CUU, CUC, CUA, and CUG. It was observed that the RSCU value for the specific amino acid increases with the number of codons. Leucine, serine, and arginine preferred six codon types, while methionine and tryptophan preferred one codon type. Similar codon usage patterns were exhibited in the three *Lespedeza* species (Table S3).

**Figure 3:**
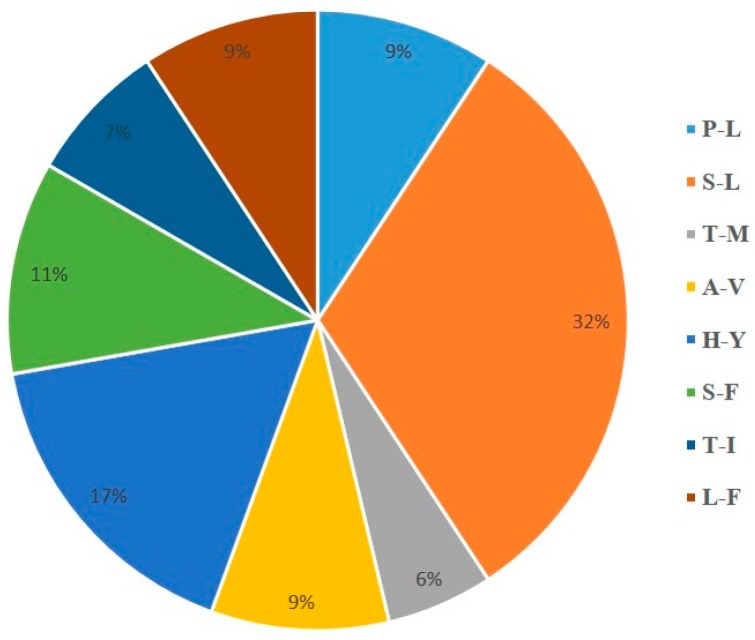
Types and percentage of amino acid exchanges arising from chloroplast RNA editing in *Lespedeza*.

All RNA editing sites were in the same nucleotide and amino acid positions in *Lespedeza* species. A total of 84 RNA editing sites were predicted in 35 protein-coding genes in cp genomes. All the codon changes were C to T conversions. The most editing sites were observed for *ndh* genes. The *ndhB* gene showed the most (13) editing events. Among the *ndh* genes, *ndhB* showed maximum editing events (13), followed by *ndhD* (12), *ndhF* (11) *ndhA* (3), and *ndhG* (3). The most abundant amino acid change was the S to L, with 18 out of 84 editing events (Figure 3). Editing was observed at first (21) and second (63) codon positions. The H to Y, P to S, and L to F amino acids conversions were due to changes in first codon position, while the P to L, S to L, T to M, A to V, T to I, and S to F changes occurred due to second codon positions (Table S4).

### 2.3. Simple Sequence Repeats and Repeat Structure Analysis

**Figure 4:**
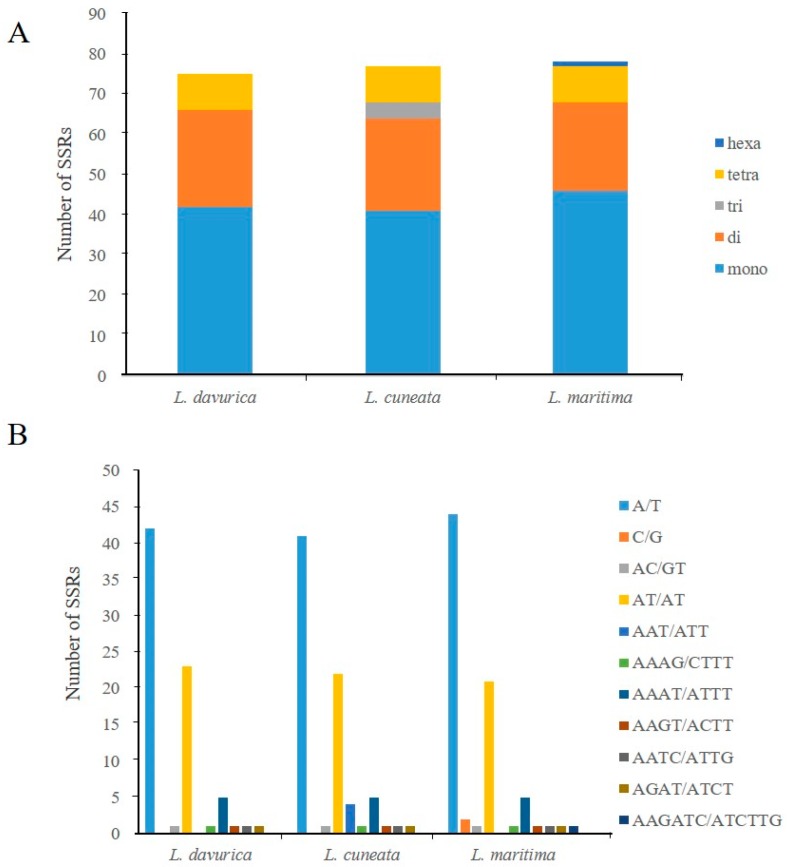
Distribution of simple sequence repeats (SSRs) in the cp genomes of *Lespedeza*. (**A**) Type of SSRs in the chloroplast genome of three species. (**B**) Distribution of SSRs in the chloroplast genome of three species.

Simple-sequence repeats (SSRs), also known as microsatellites, are short tandem repeat DNA sequences that consist of repeating 1–6 nucleotide motifs distributed throughout the chloroplast genome [31]. It shows a high degree of polymorphism that can be used as a molecular marker in species identification [32]. A total of 75, 77, and 78 SSRs were detected in *L. davurica*, *L. cuneata*, and *L. maritima* cp genomes, respectively (Figure 4A; Table S5). Mononucleotide repeat A/T was the most abundant (Figure 4B) and was encountered 42, 41, and 44 times in *L. davurica*, *L. cuneata*, and *L. maritima*, respectively. The numbers of dinucleotide SSRs (24, 23, and 22, respectively) were more than those of tetranucleotide SSRs, which were nine in each species. No pentanucleotide SSRs existed in these three species, and no trinucleotide SSRs were present in *L. davurica* and *L. maritima*. A hexanucleotide repeat was identified only in *L. maritima*. SSRs were distributed in LSC, SSC, and IR regions, with an abundance in the LSC as compared to the SSC and IR regions.

**Figure 5:**
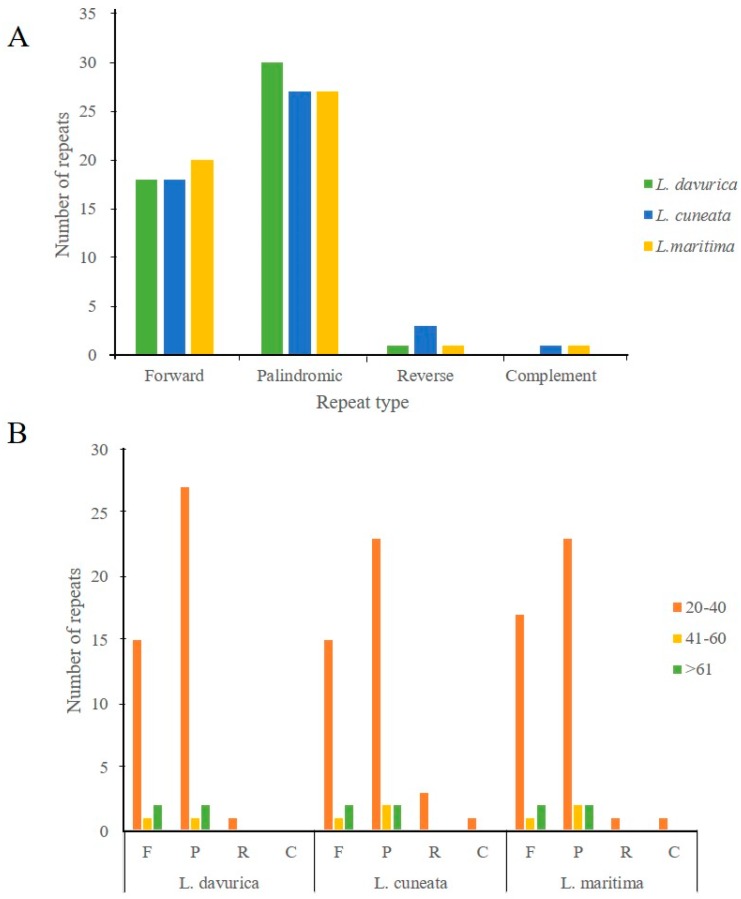
Repeat structure analysis of three *Lespedeza* sps. (**A**) Number of different types of repeats identified from the cp genomes. (**B**) Frequency of repeat lengths observed for different repeats. F, Forward; P, Palindromic; R, Reverse; C, Complement.

Dispersed repeat sequences occurred in multiple copies of the genome, and they are recognized as a source of genomic variation and rearrangement [33]. This generates diversity among genomes in a population, which is useful for studies on the phylogenetic relationships among different genomes. Most of these repeats are distributed in the intergenic spacer (IGS) and intron sequences. The structure analysis of repetitive sequences revealed an equal number of repeats in the three *Lespedeza* chloroplast genomes with slightly altered repeat types. The repeats in *L. davurica* were comprised of 18 forward, 30 palindromic, and one reverse repeats, *L. cuneata* contained 18 forward, 27 palindromic, three reverse, and one complement repeats, while *L. maritima* consisted of 20 forward repeats, 27 palindromic repeats, one reverse, and one complement repeats (Figure 5A). The palindromic type was the most abundant repeat, with lengths in the range of 20–40 bp in all the three species (Figure 5B). These repeats could provide valuable information for studying the population phylogeny of these three species.

### 2.4. Sequence Divergence Analysis

**Figure 6:**
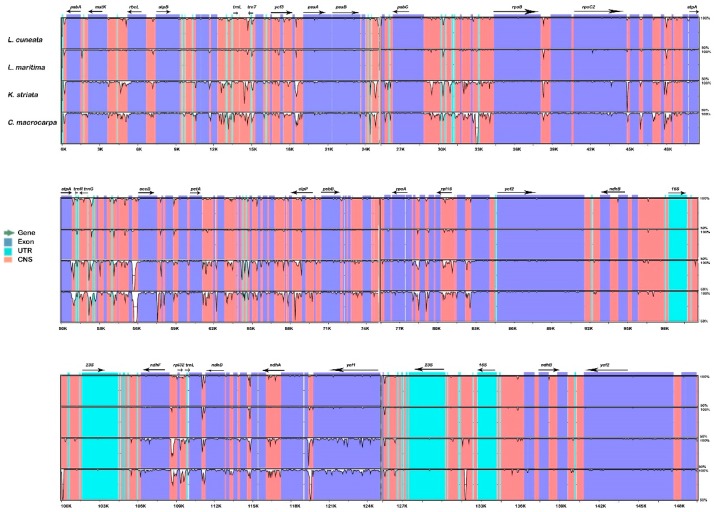
Sequence alignment plot comparing five cp genomes with *L. davurica* as a reference. Genome regions are color coded as protein coding, rRNA coding, tRNA coding, or conserved noncoding sequences. The vertical scale indicates the percentage identity, ranging from 50% to 100%.

**Figure 7:**
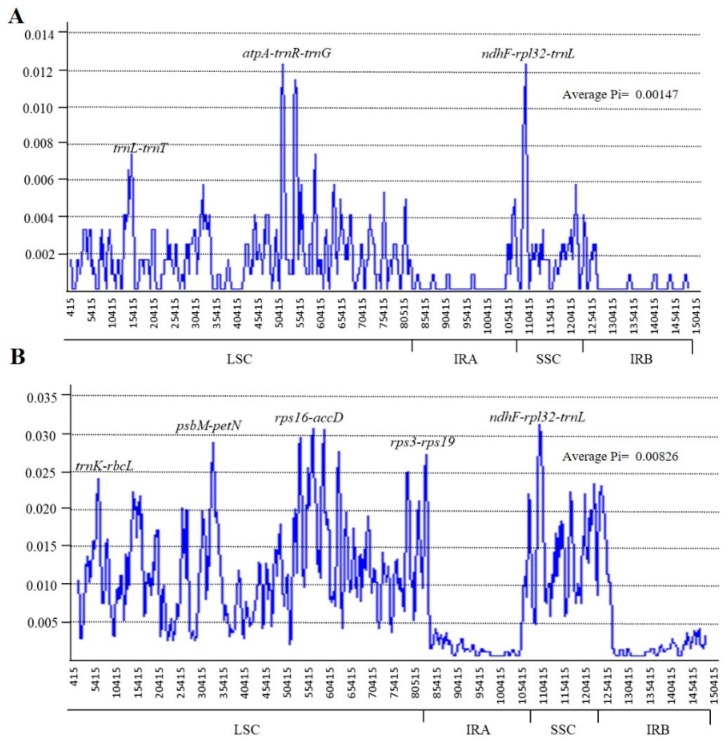
Sliding window analysis of the whole chloroplast genomes. (**A**) Pi among *Lespedeza* species. (**B**) Pi among species in *Lespedezinae*. Window length: 800 bp; step size: 200 bp. X-axis: Position of the midpoint of a window. Y-axis: Nucleotide diversity of each window.

mVISTA was used to compare whole chloroplast genome sequences of subtribe *Lespedezinae* including *L. cuneata*, *L. maritima*, *Kummerowia striata*, and *Campylotropis macrocarpa*, with *L. davurica* as a reference (Figure 6). Sequence divergence was very low among the species, suggesting a rather conserved cp genome. However, high nucleotide variations were observed between the three *Lespedeza* sequences for the protein-coding region *rpl32*, and intergenic regions such as *trnL-trnT*, *atpA-trnR-trnG*, *ndhF-rpl32*, and *rpl32-trnL*. Sliding window analysis results revealed the same variable regions in the cp genomes of the three *Lespedeza* species. The sequence divergence level was estimated through the calculation of nucleotide variability (Pi) using the DnaSP software, and the average value of Pi among the *Lespedeza* species was estimated to be 0.00147 (Figure 7A). Two highly variable regions, *atpA-trnR-trnG* and *ndhF-rpl32-trnL*, were observed with the higher Pi values (>0.01) and were located at the LSC and SSC regions, respectively. Similar results were also obtained from mVISTA. However, the average Pi value of 0.00826 among all species in *Lespedezinae* (Figure 7B) indicated the existence of divergence between cp genome sequences of different genera. The loci *trnK-rbcL*, *psbM-petN*, *rps16-accD*, *rps3-rps19*, and *ndhF-rpl32-trnL* showed variability in both analyses, and *rps16-accD* and *ndhF-rpl32-trnL* showed a remarkably higher Pi value (~0.033) among all species.

### 2.5. Phylogenetic Analysis

**Figure 8:**
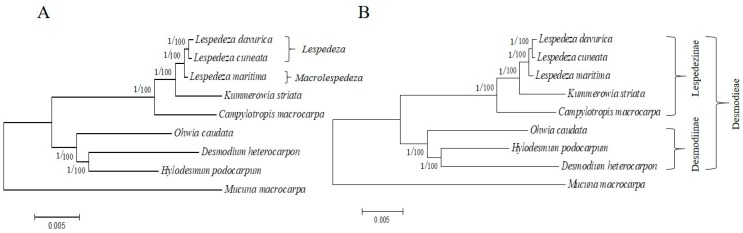
Phylogenetic relationship of Desmodieae species using maximum likelihood (ML) and Bayesian inferences (BI) analyses based on (**A**) whole chloroplast genomes, (**B**) 68 protein coding genes. The numbers in each node indicate ML bootstrap values on the right, and values on the left indicate Bayesian posterior probabilities (PP).

ML and BI trees showed similar results and identical topologies for both the datasets comprising the whole chloroplast genome sequences of different species of Desmodieae (Figure 8A) and the 68 protein-coding genes (Figure 8B). Support values in the ML tree were 100% in both datasets, and a sister relationship between *L. davurica* and *L. cuneata* was observed; moreover, both showed a sister relationship with *L. maritima*. Furthermore, *Lespedeza* formed a clade with *Kummerowia striata* and *Campylotropis macrocarpa* indicating their close relationship in the subtribe Lespedezinae. The high bootstrap and posterior probability values in this analysis suggested that the protein-coding genes and whole chloroplast genomes could be useful to find the phylogenetic positions and relationships of *Lespedeza* in the tribe Desmodieae.

## 3. Discussion

The Papilionoideae is a subfamily of Fabaceae that is the third-largest to the Asteraceae and Orchidaceae families in angiosperms. Many economically important trees and shrubs belong to this family, which comprises about 770 genera and 19,500 known species [34,35]. Among them, *Astragalus*, *Acacia*, *Indigofera*, *Crotalaria*, and *Mimosa* are the most abundant genera in this family, and they include over 3000, 1000, 700, 700, and 500 species, respectively. According to the APG III plant classification system, these genera belong to six subfamilies, Cercidoideae, Detarioideae, Duparquetioideae, Dialioideae, Caesalpinioideae, and Papilionoideae, within family Fabaceae. Papilionoideae is a widely distributed subfamily comprising about 14,000 species from 503 genera, which belong to several tribes. The Desmodieae is one of the tribes in Papilionoideae and comprises about 32 genera [36,37]. Furthermore, this tribe has historically divided into subtribes such as Bryinae, Desmodiinae, and Lespedezinae [38]. In the Lespedezinae, a close relationship between three genera *Campylotropis*, *Kummerowia*, and *Lespedeza* has been discussed previously using the floral characteristics [8].

About 40 species listed for the genus *Lespedeza* include eastern Asia species and North American species [36]. Only Asian species belong to subgenus *Macrolespedeza*, while both Asian and North American species belong to subgenus *lespedeza* within the genus *Lespedeza*. However, these two subgenera are very closely related, and species are indistinguishable. Phylogenetic analysis of Han et al. (2014) [1] showed that *L. maritima* clustered in the *Macrolespedeza* group and *L. davurica* and *L. cuneata* clustered with the *lespedeza* group.

Here, we determined the first complete chloroplast genome sequences of subgenus *Lespedeza* using the Illumina platform and deposited them in the NCBI Genbank. The lengths of complete chloroplast genomes that we obtained were similar to the cp genome sizes in most of the angiosperms that generally vary from 120 kb to 160 kb [39,40,41]. These cp genomes showed a quadripartite organization containing a pair of IRs, LSC, and SSC regions as a typical angiosperm [42], and the sequences had similar genome structures, gene orders, and gene contents. However, the higher GC content in the IR region of two sequences could be due to the presence of ribosomal RNA in this region [43]. There were no pseudogenes observed in *Lespedeza* cp genomes, whereas other angiosperms, such as *Vernicia fordii*, *Cynara humilis*, and *Jacobaea vulgaris*, showed pseudogenes in their cp genomes [44,45]. Among 83 protein-coding genes, *psbL*, *rps19*, and *ndhD* showed initiation codons other than the standard ATG in both species. *psbL* started with TTG, while *rps19* and *ndhD* started with GTG and ATC, respectively. Similar results of a GTG initiation codon in *rps19* were also reported in the tobacco chloroplast genome [46]. Further, a TTG initiation codon was reported in the chloroplast *infA* gene in the tobacco plant [47,48].

Codon usage bias reflects the importance of molecular evolutionary phenomena such as mutation, selection, and random genetic drift [27]. GC content in codon positions is one of the factors that shape the codon usage biases in different organisms. Typically, the overall GC content for a cp genome of a land plant is low due to the AT-rich intergenic regions [17]. A similar result was observed in the *Lespedeza* cp genomes. In higher plants, RNA editing occurs during the post-translation process, which means the amino acids change by converting cysteine (C) to uridine (U) at the specific codon. This is an essential mechanism for RNA maturation to avoid incorrect mutations. This process happens when PPR protein (pentatricopeptide repeats) binds to the transcript. RNA editing sites have been identified and validated in many plants including *Arabidopsis*, tobacco, pea, and tomato [29]. In our study, 84 RNA editing sites were predicted in 35 protein-coding genes of the *Lespedeza* cp genome. In *Ligularia* (Asteraceae) cp genomes, 48 RNA editing sites were reported [25]. Serine (S) conversion to leucine (L) was 22% of the editing, followed by 17% histidine (H) to tyrosine (Y) conversions. Although RNA editing pattern is conserved in studied *Lespedeza* cp genomes, detection of RNA editing sites is important to understand the missense mutations of genes.

In this study, SSR distribution was different in each species. In *L. davurica* and *L. cuneata*, SSRs were distributed in LSC and SSC region, while in *L. maritima*, C/G type SSRs in the IR. However, the majority of SSRs were located in the LSC region in all the three species. This was similar to the previously reported cp genome of *Cercis chuniana* in Fabaceae. Among the SSRs, the highly abundant mononucleotide was the A/T and dinucleotides were mostly composed of AT, suggesting that the SSRs in the cp genomes of the three *Lespedeza* species were more abundant in AT than in G/C repeats.

Based on mVISTA and sliding window analysis, we identified the sequence divergence regions that could be used as effective molecular markers. Variable regions *trnL-trnT*, *ndhF-rpl32-trnL*, and *atpA*- *trnR*-*trnG* could be ideal as molecular markers to distinguish the subgenera *Lespedeza* and *Macrolespedeza* species. The divergence was distributed in the LSC and SSC regions and mainly associated with the non-coding regions, except for *rpl32*. Previous studies have shown that the sequence divergence of the IR region was remarkably lower than that of the LSC and SSC regions in many angiosperms [49].

With the development of complete chloroplast sequencing technologies in recent decades, accurate phylogenetic studies have been enhanced, revealing the path to many unknown relationships within the plant kingdom [50]. Therefore, in this study, we used two datasets representing whole chloroplast genomes and 68 protein-coding genes that were present in all cp genomes of Desmodieae. Phylogenetic trees were constructed to determine the phylogenetic position of two *Lespedeza* (belonging to subgenus *Lespedeza*) among the species in the Desmodieae. Our results showed a close relationship between these two species and confirmed their phylogenetic position in the Desmodieae. Therefore, we suggest that whole chloroplast genomes and protein-coding genes could be used to find the phylogenetic positions and relationships of *Lespedeza* in the tribe Desmodieae. According to a previous phylogenetic analysis based on 67 protein-coding genes of cp genomes, *L. maritima* (belonging to subgenus Macrolespedeza) showed a monophyletic relationship with two genera *Campylotropis* and *Kummerowia*. Furthermore, *L. maritima* showed a sister relationship with *Kummerowia*, and these two together showed a sister relationship with *Campylotropis* [14]. Our results also revealed the same relationship between these two genera and resolved the *Lespedeza* position in the tribe Desmodieae. However, at present, complete chloroplast genomes of many *Lespedeza* have not been sequenced yet. Those data are needed to distinguish each species and for further investigation of this genus.

## 4. Materials and Methods

### 4.1. Plant Materials and DNA Sequencing

Fresh leaves of *L. davurica* and *L. cuneata* were collected from a rebuild grassland near Yan’an, Shaanxi, China (36°39′20″ N 109°24′26″ E) in July, 2018. Total genomic DNA was extracted from silica-dried leaves using the modified CTAB method [51]. Purified DNA was used for paired-end library preparation according to the manufacturer’s protocol (Illumina, San Diego, CA, USA). Genomic DNA was sequenced on the HiSeq X Ten platform (Illumina, San Diego, CA, USA). Complete cp genomes of another six Desmodieae species, namely, *Lespedeza maritima* (NC_044115), *Kummerowia striata* (MG867569), *Campylotropis macrocarpa* (MG867566), *Hylodesmum podocarpum* (MG867568), *Desmodium heterocarpon* (MG867567), and *Ohwia caudate* (MG867572) were downloaded from Genbank.

### 4.2. Genome Assembly and Annotation

High-quality reads were obtained by trimming the raw reads using the Trimmomatic tool [52] and then assembled using the GetOrganelle pipeline (https://github.com/Kinggerm/GetOrganelle/blob/master/get_organelle_from_reads.py). Subsequently, the assembly was polished by mapping raw reads back to the retrieved sequence, and the consensus sequence was generated using Geneious 10.0.5 software (http://www.geneious.com). We confirmed the low coverage with ambiguous regions by mapped to the reference *L. maritima*. The consensus sequence was further adjusted by aligning with the available complete cp genomes of Desmodieae. Annotation of all protein-coding sequences and tRNA and mRNA genes were performed using the Dual Organellar GenoMe Annotator (DOGMA) with manual corrections of the uncertain annotations [53]. tRNA genes were confirmed with the tRNAscan-SE program [54]. To determine the accuracy of assembled complete cp genomes, we carried out BLAST using the available sequences of *L. davurica* and *L. cuneata* in the NCBI database. We found that these sequences were highly matched with our assembled cp genomes. The circular cp genomes of *Lespedeza* were drawn using the OrganellarGenome DRAW tool (OGDRAW) [30]. Complete chloroplast genomes of *L. davurica* and *L. cuneata* were deposited in Genbank with the accession numbers MH800328 and MN268503 respectively.

### 4.3. Codon Usage Bias, RNA Editing Sites, and Sequence Divergence

Relative synonymous codon usage (RSCU) values and GC contents of protein-coding sequences were calculated by MEGA 6.0 [55]. RNA editing sites of 35 coding sequences of the *Lespedeza* species were predicted by the online PREP-Cp program with a cutoff value of 0.8 [56]. The mVISTA program [57] was used to align the whole chloroplast genomes of subtribe Lespedezinae. Divergent regions of the chloroplast genomes were determined and variation of nucleotide diversity (Pi) was calculated using the sliding window using DnaSP v5.10. [58] with 200-bp step size and 800-bp window length, which were the common parameters reported in the literature [59,60,61].

### 4.4. Repeat Sequences Analysis

The MISA [62] microsatellite finder was used to detect SSRs with the parameters set to 10 repeat units for mononucleotide SSRs, 5 repeat units for dinucleotide SSRs, 4 repeat units for trinucleotide SSRs, and 3 repeat units each for tetra-, penta-, and hexanucleotide SSRs. To identify the forward, palindromic, reverse, and complement repeats in three species, REPuter [63] was used.

### 4.5. Phylogenetic Analysis

The whole chloroplast genome sequences of six species belonging to tribe Desmodieae and the out-group *Mucuna macrocarpa* were downloaded from the NCBI database to determine the phylogenetic positions of the two *Lespedeza* species analyzed in the present study. Two datasets (complete chloroplast genomes and 68 concatenated protein-coding genes) were used for the analysis. These 68 genes were conserved in all nine cp genomes. Coding sequences extracted from each cp genome were aligned using the MAFFT [64] alignment under default parameters, and these alignments were concatenated using Geneious software. Phylogenetic analysis was performed by the maximum likelihood (ML) and Bayesian inference (BI) methods using the final sequence alignments of both datasets. ML trees were constructed with MEGA 6.0 based on the general time reversible (GTR) + I + G model with 1000 replicates. A discrete Gamma distribution was used with four categories. Initial tree(s) for ML were obtained by the neighbor-joining and BioNJ algorithms. BI analysis was conducted using MrBayes 3.2 [65] and a GTR substitution model was used. Two independent Markov chain Monte Carlo (MCMC) chains were run for 1.5 million generations with a subsampling frequency of 200 generations.

## 5. Conclusions

The study presented the complete chloroplast genomes of *Lespedeza davurica* and *L. cuneata*. This is the first study reporting the cp genome sequences of *Lespedeza* subgenus. Our results showed that gene contents, orders, and structures were highly conserved among these species. Results based on codon usage bias and predicted RNA editing sites are useful for further studies about this genus. Our results based on the sequence divergence analysis identified variable regions that can be used for identifying *Lespedeza* species. Finally, the data obtained from this study could provide a useful resource for further research on these species at the genomic scale and in establishing deeper branches of the phylogeny.

## Figures and Tables

**Table 1:** Summary of chloroplast genome features of *Lespedeza*.

Characteristics	*Lespedeza davurica*	*Lespedeza cuneata*
Accession Number	MH800328	MN268503
Total cpDNA Size	149,010	149,010
LSC	82,373	82,395
IR	23,831	23,832
SSC	18,975	18,951
Total CDS Length	77,868	77,868
Total tRNA Length	2794	2794
Total rRNA Length	9062	9062
Total Number of Genes	128	128
Coding Genes	83	83
rRNA Genes	8	8
tRNA Genes	37	37
Total GC %	35.0	35.0
LSC	32.5	32.5
IR	42.2	42.2
SSC	28.1	28.1
CDS	36.0	36.0

LSC: Large single copy, IR: Inverted repeat, SSC: Small single copy, CDS: Coding sequences, GC: Guanine and Cytosine.

## Supplementary Materials

The following are available online at https://www.mdpi.com/2223-7747/9/1/51/s1, Table S1: Summary of gene contents present in the *Lespedeza* chloroplast genomes, Table S2: Comparison of chloroplast genomes among Desmodieae, Table S3: Summary of relative synonymous codon usage (RSCU) of the codon usage in the *Lespedeza* cp genomes, Table S4: Predicted RNA editing sites in chloroplast protein-coding genes of *Lespedeza*, Table S5: Distribution of Simple sequence repeats (SSRs) in the three *Lespedeza* cp genomes.
